# A Preliminary Investigation of a Specialized Music Therapy Model for Children with Disabilities Delivered in a Classroom Setting

**DOI:** 10.1155/2016/1284790

**Published:** 2016-11-23

**Authors:** Jenna Mendelson, Yasmine White, Laura Hans, Richard Adebari, Lorrie Schmid, Jan Riggsbee, Ali Goldsmith, Burcu Ozler, Kristen Buehne, Sarah Jones, Jennifer Shapleton, Geraldine Dawson

**Affiliations:** ^1^Department of Psychology, The University of North Carolina at Greensboro, Greensboro, NC, USA; ^2^Department of Psychiatry and Behavioral Sciences, Duke University, Durham, NC, USA; ^3^Education and Human Development Incubator and Social Science Research Institute, Duke University, Durham, NC, USA; ^4^Program in Education, Duke University, Durham, NC, USA

## Abstract

Music therapy is gaining popularity as an intervention strategy for children with developmental disabilities, including autism spectrum disorder (ASD). This study was a pilot investigation of a classroom-based music-based intervention,* Voices Together®*, for improving communication skills in children with ASD and children with intellectual disabilities. Four local public elementary school special education classrooms, serving 5 children with a classification of autistic disorder and 32 children with intellectual disability without autism, were randomly selected to receive one of two levels of exposure to* Voices Together* music therapy: “long-term” (15 weeks beginning in January 2015 (Time 1), *n* = 14) or “short-term” (7 weeks beginning 7 weeks later in February (Time 2), *n* = 17). Using observational ratings, investigators reliably scored participants live in terms of their level of verbal responsiveness to prompts during three songs featured each week of the program. Both groups demonstrated increases in verbal responses over time; however, only the long-term group demonstrated significant within-group increases. Preliminary findings suggest that music therapy delivered in a classroom in 45-minute weekly sessions for 15 weeks can promote improvements in verbal responsiveness among individuals with autism and other developmental disabilities. Findings warrant further investigation into the efficacy of classroom-based music therapy programs.

## 1. Introduction

Over the last 12 years, the prevalence of developmental disabilities in US children has increased by 17.1% [1]. Students with developmental disabilities often have challenges in the areas of communication and social/emotional learning, which can lead to social isolation, problematic behaviors, and deficits in academic learning [2]. However, individuals with developmental disabilities have been found to respond well to the incorporation of music in intervention programs, in terms of both gains in communication skills and stronger social functioning [3], suggesting that music therapy may present an effective means of improving communication and social skills in this population. The American Music Therapy Association (AMTA) defines music therapy as “the clinical and evidence-based use of music interventions to accomplish individualized goals within a therapeutic relationship by a credentialed professional who has completed an approved music therapy program” [4].

Early research examining the efficacy of music therapy for improving communication and social skills for children with developmental disabilities suggested that it may be an effective approach for enhancing nonverbal and verbal communication, as well as social engagement [3]. Based on these findings, the AMTA lists improvements in communication skills as among the primary functions of music therapy interventions [4]. Music has been found to be an effective tool in the promotion of communication and social skills among children with developmental disabilities, including those with and without ASD (e.g., [5, 6]), and with varying levels of symptom severity, including nonverbal children with ASD (e.g., [7]). Moreover, incorporation of music therapy with other intervention strategies in the classroom has been found to positively impact social skills among children with a range of developmental disabilities [8].

Buday [9] conceptualized the patterns presented in musical compositions as a potential mechanism for this efficacy. Patterns in music have been theorized to help hold children's attention and promote language development [10]. Moreover, children with ASD and intellectual disability (ID) have been found to be more likely to attend to auditory than visual cues when the auditory stimulus is musical [11], supporting the hypothesis of music as an effective vehicle for intervention through its unique ability to hold children's attention. Positive affective responses that occur with music have also been hypothesized to increase participation in therapy, as well as progress toward goals (Buday, 1995). Moreover, music has been described as an inherently social activity and has been directly linked to social interaction [12].

Further supporting the potential efficacy of music-based interventions for individuals with developmental disabilities, research has demonstrated that individuals with developmental disabilities, including those with ASD, process information in unique ways, which can be manifested in relatively enhanced musical abilities [13]. For example, individuals with ASD have been found to respond emotionally to music in a way that is similar to neurotypical individuals [14]. Music abilities are not limited to individuals with ASD who demonstrate exceptional musical skills [15]. Stanutz and colleagues [13] demonstrated that even those children with ASD who had no previous musical training possessed better pitch discrimination abilities and superior long-term memory for melody, compared to IQ- and age-matched neurotypical controls. Given the centrality of communication and social deficits among children with developmental disabilities [2, 16], therapists may be able to leverage the unique ability of music to hold children's attention and increase participation in therapy while promoting positive affect and social interactions in order to help children with developmental disabilities improve their communication and social skills.

Taken together, music therapy may be an excellent fit for the therapeutic needs of individuals with developmental disabilities, especially given research indicating its impact on communication and social skills, which are among the core deficits of ASD and are also implicated in a range of other developmental disabilities [2, 16]. Perhaps for these reasons, music is already commonly incorporated into therapeutic interventions of children with ASD, featuring in some capacity in as many as 12% of classroom-based interventions [17]. However, to date, very few controlled studies exist that examine the feasibility and efficacy of music-based interventions in a classroom setting.

This project was designed to be a pilot study of a classroom-based music therapy program,* Voices Together*, on the social and communication skills of children with developmental disabilities. Pilot studies have been determined to be a key component to the research process as a precursor to randomized controlled trials (RCTs; [18]). Pilot studies can be valuable tools in determining the feasibility of participant recruitment in completion of the research protocol [19] prior to conducting a full-scale RCT. However, while pilot studies may provide some early support for research hypotheses, findings must be interpreted with caution [18].

The* Voices Together* program is a proprietary and specialized music therapy model that integrates music and multisensory experiences to teach communication and social/emotional skills to people with intellectual and developmental disabilities. Among the core methods employed in the program is VOICSS™: Vocal Interactive Communication and Social Strategies. This method uses structured music-based interactions to help participants develop their skills in domains of communication and social/emotional learning.

The first hypothesis of this study was that participation in the* Voices Together* program would lead to increases in social and communicative behavior both in terms of behavioral observations made during participation in the* Voices Together* program and in terms of general classroom participation (as measured by teacher ratings). Secondly, while increases in social behavior were expected in both groups, improvements were expected to be more robust in the long-term group. Thirdly, because all songs in the* Voices Together* program employ comparable strategies to evoke social and communicative responses, no differences in improvements to social and communicative responses were hypothesized across the three songs employed in this project.

## 2. Method

### 2.1. Participants

Four special education classrooms (two Kindergarten-2nd grade and two grades 3–5 classrooms) in the Durham Public Schools participated in this study. Thirty-three children, including 5 children with ASD and 32 with intellectual disability without ASD, participated. Diagnosis was based on the school system classification of the children. Communication skills of students, as assessed by qualitative teacher report, were also variable, ranging from minimally verbal to conversational speech. Because participants were selected directly by the school district, information pertaining to age, IQ, symptom severity, gender, and the specific classroom placements of the 5 children with ASD was not made available to the researchers.

### 2.2. Procedure

Within each level of classroom (Kindergarten-2nd grade versus 3rd–5th grade), classrooms were randomly selected to receive one of two levels of exposure to* Voices Together* music therapy: “long-term” (15 weeks beginning in January 2015 (Time 1), *n* = 14) or “short-term” (7 weeks beginning 7 weeks later in February (Time 2), *n* = 17).

#### 2.2.1. *Voices Together* Program

*Voices Together* is a specialized music therapy model designed to encourage communication and social behavior. Technique strategies and songs are interactive by design and inherently have a high expectation of a reciprocal response by establishing a pattern of prompt-response as part of each song. These strategies and songs are geared to initiate and maintain conversation, while exploring emotional, behavioral, and conceptual areas critical to social connection. The trained and licensed music therapist uses vocal exchange, a social/emotional curriculum, and musical instruments all geared toward eliciting social and communicative responses from participants.* Voices Together* is based on the VOICSS (Vocal Interactive Communication and Social Strategies) approach, which aims to increase social and communication outcomes by using program songs aimed at encouraging social interaction and verbal responses. The basic structure of the songs within the VOICSS method, which is unique to the* Voices Together *program, follows this pattern:

1. Brainstorming and providing written responses on a whiteboard for the group on a predetermined topic (VT curriculum) that supports the social/emotional and communication objectives
2. Choosing a peer leader for the song who facilitates the song with support from the therapist
3. Singing and speaking; reciprocal and simple metered patterns
4. Listening
5. Responding
6. Repeating and reinforcing responses by group

Songs incorporate specific techniques to make communication more likely, including leaving requests for a response on an unresolved musical note, internal structure for turn-taking that alternates between group and individual response, and directly asking each individual questions that alternate between singing and speaking. Therapists also regularly employ a strategy known as “responsive prompting” (consisting of prompts for communication that vary in intensity based on each participant's ability level) to encourage verbal responses, both within the structure of program songs and during spontaneous group interactions. To ensure fidelity to the treatment protocol,* Voices Together* providers are observed and rated across 7 domains: (1) providing appropriate energy to engage participants, (2) facilitating peer-to-peer awareness and connection, (3) facilitating client self-awareness, (4) facilitating and adapting appropriate speech/communication goals, (5) facilitating emotional awareness, (6) modifying techniques based on participant ability, and (7) appropriate support of self-management of behaviors.

Three* Voices Together* songs were selected for the purpose of this study: the “*Hello*” song, which comprises an introduction to the session and requires participants to greet each other and each say their name as part of the song; the “*Feelings*” song, which requires participants to respond to group by describing how they are feeling that day; and the “*Topic*” song, which requires participants to respond to song lyrics by bringing up a topic of interest with the group. All three songs use VOICSS strategies to evoke social responses from participants and are designed to improve social and communication skills by encouraging participants to generate novel social and communicative responses within the structure of a familiar format.

A licensed music therapist trained in the* Voices Together* program led all therapy sessions. As per the* Voices Together* curriculum, participants sat in a horseshoe shape in the classroom, with the music therapist sitting centrally, facing the students. Students participated in a single 45-minute session of* Voices Together* once weekly for 15 weeks in the long-term group and 7 weeks in the short-term group.

### 2.3. Dependent Variables

#### 2.3.1. Behavioral Observations

Four advanced level undergraduate students and three research staff rated students' behavior, focusing on communication. Raters were trained to code both student and therapist behavior during twice-weekly coding meetings led by one of the coinvestigators of this project during the month leading up to the first session. The coding procedure was designed specifically for the aims of this project, and each participant was coded individually. Coding was completed during live observations that took place in the classroom during the therapy sessions at each of the three time points. Two raters attended each coding session, coded independently of each other to calculate reliability, and then adjudicated differences through discussion at the end of the session. Although videotaped coding using a standardized coding procedure was initially planned for this project, a live-coding strategy was ultimately employed due to difficulty obtaining consent from parents of all participants to use video recordings of their children in the classroom.

Both therapist prompts and child responses were coded. Therapist prompts were reverse-scored such that the lowest level of prompting (representing the highest level of spontaneity in participant response) received the highest score. Therapists' level of prompt required to elicit a response was coded, with prompts ranked from the least directive (pause alone) to the most directive (full verbal modeling). Visual prompts were initially included among the coded prompts but dropped when it became apparent that they were used systematically in one classroom and as such were not reflective of participant ability level. Each participant was coded individually. If a participant was absent at the time of a scheduled behavioral observation, the observation was rescheduled for a time during the week when all participants could be present. There was one instance where this was not possible, and the absent participant's data was not included in statistical analyses.

Participants' levels of verbal and social responses were coded on a Likert scale of 1–8 for each domain. Responses were numerically ranked from the least social/verbal (8 = body orientation/no response) to the most social/verbal (1 = full sentence verbal response). Prompts were scored on a similar Likert rating scale ranging from 1 (pause alone) to 5 (hand-over-hand). Lower mean score prompts indicate less intrusive prompts. Scores for the prompts and requisite responses for all three songs were combined to form the composite “*All Songs*” score. Lower mean scores indicated more positive responses. These scores were then summed to provide a total overall score for social/verbal responsiveness during the observation for each participant. Thus, scores closer to 0 are indicative of* higher* levels of social and verbal interaction. Given previous research suggesting that music may be an effective mode of therapy based in part on the promotion of positive affect (Buday, 1995), participants were also coded for positive affect, defined simply as whether or not the participant smiled over the course of the observation.

Reliability was calculated by the intraclass coefficient (ICC). ICC was selected as an appropriate measure of interrater reliability for this project because it allows for calculation of overall reliability for teams of larger than three coders and incorporates into reliability calculation the magnitude of disagreement (as opposed to an all-or-nothing agreement, such as what is calculated by Cohen's kappa; [20–22]). Because a series of pilot videos were coded by multiple coders as a measure of overall reliability, the single-measure ICC is reported. Based on general guidelines delineated by Cicchetti [23], reliability is considered fair when the ICC is between .40 and .59, good when the ICC falls between .60 and .74, and excellent when it is between .75 and 1.0. An ICC of 1.0 represents perfect interrater agreement. The ICC was used to calculate overall reliability for the prompts, responses, and positive affect coding across three training videos featuring implementation of the* Voices Together* program in a classroom setting, very similar to the* in vivo* coding completed by the coding team. For the prompt coding, the ICC ranged from fair to excellent across the three training videos (the full range is reported here; ICC_(2,1)_ = .52–.80). Reliability for coding responses also ranged from fair to excellent across the three videos (ICC_(2,1)_ = .52–.97), and reliability for positive affect was slightly poorer, in the fair range across the three training videos (ICC_(2,1)_ = .42–.54).

#### 2.3.2. Teacher Ratings

Teachers completed the Social Skills Improvement System-Rating Scale (SSIS-RS; [24]), an 83-item scale designed to assess the efficacy of interventions targeting social skills and to demonstrate both social and intervention validity [25]. The SSIS-RS has been found to have strong psychometric properties across both the Social Skills and Problem Behaviors subscales, including high internal consistency and test-retest reliability [26]. The SSIS-RS Teacher rating survey consists of 13 subscales encompassing social skills, problem behaviors, autism spectrum behaviors, and academic achievement. Social skills, problem behaviors, and autism spectrum behaviors were rated using a 4-point Likert scale labeled “Never,” “Seldom,” “Often,” and “Almost Always.” Additionally, the academic achievement subscale uses a 5-point Likert scale for the participant's percentile rank in the classroom from “Lowest 10%” to “Highest 10%.” The scale takes 20–25 minutes to complete and is used to evaluate social skills and competing problem behaviors such as communication, self-control, bullying, hyperactivity, and autism spectrum behaviors. For the purposes of this study, main analyses focused on the Assertion, Communication, and Engagement subscales, as these were hypothesized to be most closely related to the goals of the therapy program.

Teachers completed the SSIS-RS about each participant at Time 1 (the first week of the program for the long-term group), Time 2 (7 weeks into the program, the starting point for the short-term group), and Time 3 (15 weeks into the program). Teachers were compensated with $200.00 after completing the SSIS-RS at Time 3. A new teacher was hired in one of the two short-term classrooms at Time 2 of the study. To account for possible differences in reporting due to the second teacher's new status in the classroom at Time 2, both the new teacher and the previous teacher completed the questionnaire at Time 2 and scores were averaged. The new teacher then completed questionnaires independently at Time 3. Given the necessity of organizing the classroom schedule in order to hold therapy sessions during the school day, teachers were not blind to whether participants were in the short- or long-term groups.

## 3. Results

To assess improvements in social and communicative behavior, data were analyzed using a series of repeated measures ANOVAs, first to assess increases in behavioral observations of responsiveness and next to assess increases in the level of prompt used (level of prompt was reverse-scored such that increased scores represent more the use of more minimal prompts). Scores from individual participants in each classroom were then averaged according to classroom to provide a single score for each participating class. The one participant who was not present for one of the behavioral observations was excluded from this average and from all subsequent analyses. Separate repeated measures ANOVAs were run for each song (*Hello*,* Feelings*, and* Topic*), as well as one for combined observations from all three songs (*All Songs*) and one to assess change in terms of positive affect. Where ANOVAs demonstrated significant differences across time points, within-group paired* t-*tests were run to examine more specifically whether changes occurred in both the short- and the long-term groups. Lastly, a series of paired* t-*tests were run to examine improvements in social skills as rated by teachers on the SSIS-RS. In cases where live-coders provided discrepant responses, results using the averaged scores from both coders are reported here.

### 3.1. Behavioral Observations

**Figure 1:**
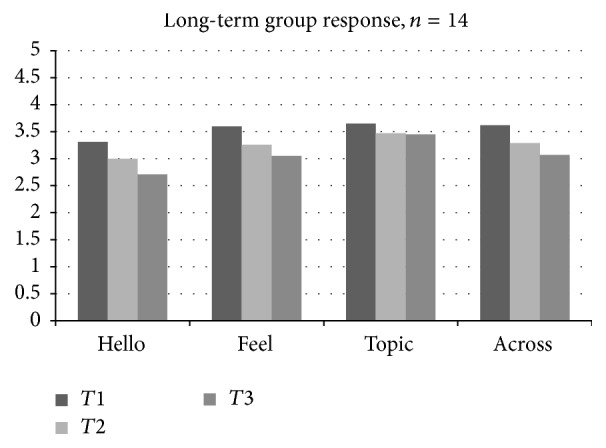
Long-term group response across time points.

To assess hypothesis 1 (that both groups would demonstrate improvements in the observed level of social and communicative response over time), groups were combined and levels of prompts and responses were examined across both the short- and the long-term groups from* T*2 to* T*3. A repeated measures ANOVA examining the level of response across both groups over time indicated a significant increase in level of response to the* Feelings* song from Time 1 to Time 3 (Figure 1;* Feelings response*, *F*(2,28) = 3.32, *p* < .05). Follow-up paired* t*-tests using combined scores from both groups indicate higher level responses across time points for the* Feelings* song (*t*(29) = 1.77, *p* = .09,* Hello* song,* t*(29) = 1.78, *p* = .08, and trending significance in* All Songs t*(29) = 1.91, *p* = .0.07; see Figure 3). A closer examination, however, indicates that while these improvements were a trend in the short-term group, they were only significant in the long-term group. The long-term group demonstrated significant increases in response to the* Feelings* song between Time 1 and Time 2 (*t*(14) = 2.63, *p* < .05). The long-term group also demonstrated a significant increase in average level of response across all* Voices Together* songs from Time 1 to Time 2,* t*(14) = 2.47, *p* < .05, and between Time 1 and Time 3,* t*(14) = 2.46, *p* < .05. Additionally, the long-term group demonstrated a significant increase in average level of response across all* Voices Together* songs from Time 1 to Time 2,* t*(14) = 2.47, *p* < .05, and between Time 1 and Time 3,* t*(14) = 2.46, *p* < .05. Neither group demonstrated significant improvements in terms of level of response to the* Topic* song, or in terms of the expression of positive affect during therapy sessions. Although the short-term group demonstrated increases in level of response from* T*2 to* T*3, these improvements were not statistically significant. Overall, the first hypothesis (that both groups would demonstrate significant improvements in terms of level of response) was not supported at a statistically significant level, although the short-term group did demonstrate improvements that were not statistically significant. However, hypothesis 2 (that improvements would be more robust in the long-term group) was supported in terms of the* Feelings* song,* Hello* song, and* All Songs.*

**Figure 2:**
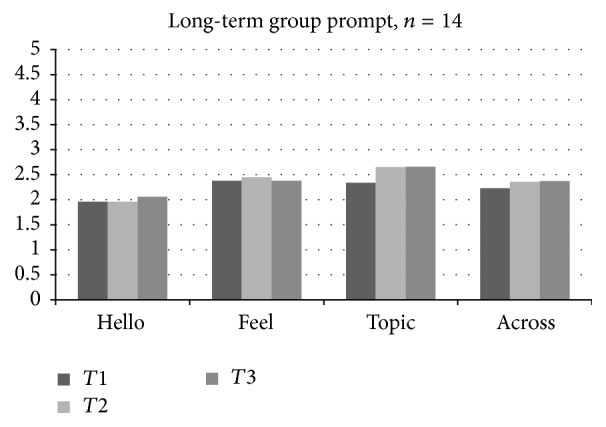
Long-term group prompt scores across time points.

**Figure 3:**
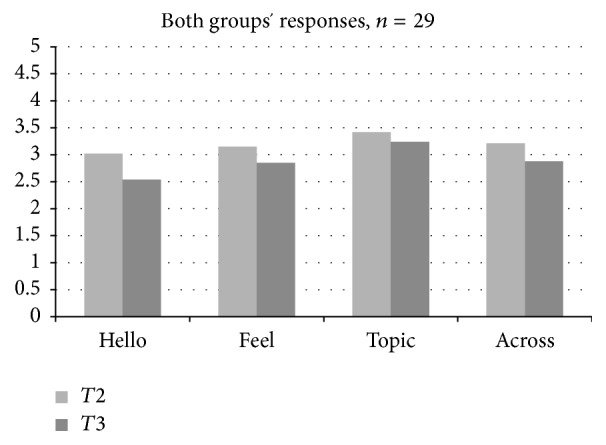
Combined responses across time points.

A repeated measures ANOVA across time points indicated differences in average long-term group mean scores for level of* Topic prompt* (the level of prompt required during the Topic song; *F*(2,28) = 3.32, *p* < .05; Figure 2). Follow-up paired* t-*tests were conducted to determine changes in mean prompt levels across the time points for each group. Unexpectedly, paired* t*-tests indicated significant* increases* in level of prompt needed during the Topic song in the long-term group from Time 1 to Time 3 (*t*(14) = 2.55, *p* < .05). Classroom observations suggest that this may be due to increasing difficulty of the* Topic* song over time. Paired* t-*tests indicate no statistically significant differences in level of prompts used between time points Time 2 and Time 3 for the short-term control group. Overall, groups did not differ significantly for prompts at Time 2 or Time 3, or in terms of level of prompt required for any other* Voices Together* song. Thus, the hypothesis of no differences in terms of improvements in behavioral observations across songs was actually* not* supported, in that the long-term group demonstrated an unexpected increase in terms of level of prompt required for the* Topic* song.

Although information regarding individual participant age was unavailable for this project, follow-up analyses were run using grade level as a proxy for participant age. As might be expected due to developmental level, participants in grades 3–5 demonstrated a higher level of response and required fewer prompts across all time points and songs than those in grades K-2. Follow-up subsets of paired* t-*tests for all songs between Time 2 and Time 3 revealed comparable improvements across both grades K-2 and 3–5 in terms of prompts during the* Hello* song (K-2:* t*(15) = 2.54, *p* = .023; 3–5* t*(13) = 3.03, *p* = .01). However, only grades 3–5 demonstrated significant improvements in terms of responses to the* Feelings* song (*t*(13) = 2.55, *p* = .024).

### 3.2. Teacher Questionnaires

Increases were predicted across the Assertion, Communication, and Engagement subscales of the SSIS-RS for both groups while participating in the* Voices Together* program. Paired *t*-tests were run on all subscales, focusing primarily on assertion, communication, and engagement for both long- and short-term groups. Contrary to the hypotheses, paired* t*-tests comparing scores from within each group across time points indicated no statistically significant differences between time points for either the long- or the short-term group on any of the SSIS-RS subscales. Thus, neither hypothesis 1 nor hypothesis 2 was supported by teacher ratings on the SSIS-RS.

## 4. Discussion

Significant improvements in terms of responses found in the long-term group, but not the short-term group, suggest that longer duration of exposure to* Voices Together* may promote a greater degree of improvement. Further research in the form of an RCT examining dosage effect of* Voices Together*, including the impact of longer or more frequent sessions, smaller versus larger groups, and time period over which the program is administered, is warranted to determine whether a greater degree of exposure to* Voices Together* promotes improvement in social and communicative behavior.

Unexpectedly, findings varied not only in terms of prompts versus responses, but also with regard to each* Voices Together* song. The* Topic* song in particular, which required participants to respond to musical prompts by bringing up a topic of interest with the group, did not appear to elicit improved responses from participants and resulted in an* increasing* level of prompts from therapists over time, counter to hypotheses. This may be due to the increasing complexity of topics expected from participants as they became familiar with the song, or to the more open-ended nature of the required response than with the* Feelings* or* Hello* song. Notably, the more structured nature of the* Feelings* and* Hello* songs may have allowed participants to rely more heavily on the musical pattern to generate a response. Overall, this finding suggests that further research as to the impact of specific song styles and musical patterns on level of communicative response from participants in music therapy programs is warranted. Also bearing consideration, songs were presented in the same order each session, suggesting that these differences may be attributable to the order of presentation rather than characteristics of the songs themselves. Counterbalancing song presentation as part of an RCT will be better able to assess the relative efficacy of each song and contribute to the understanding of mechanisms of efficacy for the* Voices Together* program.

While behavioral observations indicated significant improvement in response to* Voices Together* songs in the long-term group, these improvements were not reflected in teacher ratings on the SSIS-RS. Findings may have been obstructed by variability in rating tendencies between teachers, as well as the potential of a ceiling effect among participants who received higher scores on the SSIS-RS at baseline. Future studies may benefit from inclusion of the SSIS-RS parent report form as a means of obtaining perspectives of multiple raters for each participant as well as assessing generalization of findings outside of the school setting.

Importantly, this study was intended as a pilot study to determine the feasibility of both the research protocol and the* Voices Together* intervention itself in a public school setting. As a result, methods were subjected to significant limitations that can be used to inform methodology in future iterations of this project. Perhaps most notably, individual information about participants (including age, IQ, and diagnostic status) was not available. The lack of this information represents a major limitation of this study in terms of its ability to respond to hypotheses. Exploratory analyses using grade as proxy for age may suggest that participant age can impact the efficacy of the program, although this data should be interpreted with caution. Future RCTs of the* Voices Together* will need to ensure that demographic information for each individual participant can be made available in the selected school setting, most likely through a more lengthy and detailed consent process that was not possible as part of this project. Inclusion of relevant individual information for each participant will allow for analysis of possible moderation and mediation effects of individual characteristics, including age. Overall, however, findings from this study are promising in terms of both the feasibility and the efficacy of music-based intervention in special-needs classrooms and point to the value of a full-scale RCT to examine the impact of music-based therapy on individuals with different diagnostic classifications and individual characteristics.

Notably, again due to challenges with the consent procedure in the specific school setting, coding of behavioral observations from this project was limited to live-coding, which restricted the coding scheme to more broad-stroke observations for the sake of reliability and represents a limitation of this study. Future studies that are able to implement more detailed coding systems will allow for more nuanced and fine-tuned scoring of behavioral observations. Along these lines, video coding that allows for incorporation of more advanced behavioral coding technology may facilitate scoring of more subtle social and communicative behaviors that may not have been captured by this study.

Finally, while this study attempted to capture dosage effect by including long- and short-term treatment groups, it did not include a true control group. Future research using a randomized controlled design is warranted to examine whether the* Voices Together* program is truly effective relative to treatment-as-usual. Moreover, this project was limited to a relatively small sample size, and school selection of participants resulted in a diagnostically variable sample on whom limited demographic information is available. Thus, while these findings are promising, RCTs involving larger, better characterized samples will be key to determining whether classroom-based music therapy merits categorization as an efficacious or possibly efficacious treatment for children with developmental disabilities.

## 5. Conclusions

Findings from this preliminary investigation of* Voices Together* in the classroom indicate that children receiving 15 weeks of intervention show significant increases in level of communicative response from Time 1 to Time 3, whereas the short-term group receiving only 7 weeks of therapy showed improvements that were not statistically significant. These results lend tentative empirical support to the growing case for music therapy as an efficacious treatment in the promotion of social and communication skills among individuals with developmental disabilities. Notably, these increases were observed following 45 minutes weekly of classroom-based music therapy administered over 7 weeks and continued to increase over the 15 weeks of treatment, suggesting that* Voices Together* may be able to have a positive impact on communicative responses even in relatively small doses delivered in a classroom setting. However, teacher ratings of social and communicative behavior did not reveal significant improvements in overall level of social and communication skills in the broader classroom settings. Findings and limitations of this preliminary investigation can be used as a basis for further research into the efficacy of the* Voices Together* program in the promotion of social and communication skills among children with developmental disabilities.
